# Emergency care surveillance and emergency care registries in low-income and middle-income countries: conceptual challenges and future directions for research

**DOI:** 10.1136/bmjgh-2019-001442

**Published:** 2019-07-29

**Authors:** Hani Mowafi, Christine Ngaruiya, Gerard O'Reilly, Olive Kobusingye, Vikas Kapil, Andres M Rubiano, Marcus Ong, Juan Carlos Puyana, AKM Fazlur Rahman, Rashid Jooma, Blythe Beecroft, Junaid Razzak

**Affiliations:** 1 Department of Emergency Medicine, Yale University School of Medicine, New Haven, Connecticut, USA; 2 Department of Epidemiology & Preventative Medicine, Monash University, Clayton, Victoria, Australia; 3 Department of Disease Control & Environmental Health, Makerere University School of Public Health, Kampala, Uganda; 4 Center for Global Health Leadership, Centers for Disease Control and Prevention Center for Global Health, Atlanta, Georgia, USA; 5 Department of Neurosurgery, Universidad El Bosque, Bogota, Colombia; 6 Department of Emergency Medicine, Duke-NUS Medical School, Singapore, Singapore; 7 Department of Surgery, University of Pittsburgh School of Medicine, Pittsburgh, Pennsylvania, USA; 8 Center for Injury Prevention Research, Dhaka, Bangladesh; 9 Department of Neurosurgery, Aga Khan University Medical College Pakistan, Karachi, Sindh, Pakistan; 10 CGHS, John E Fogarty International Center, Bethesda, Maryland, USA; 11 Emergency Medicine, Johns Hopkins University, Baltimore, Maryland, USA

**Keywords:** emergency care, lmics, surveillance, registries, research

## Abstract

Despite the fact that the 15 leading causes of global deaths and disability-adjusted life years are from conditions amenable to emergency care, and that this burden is highest in low-income and middle-income countries (LMICs), there is a paucity of research on LMIC emergency care to guide policy making, resource allocation and service provision. A literature review of the 550 articles on LMIC emergency care published in the 10-year period from 2007 to 2016 yielded 106 articles for LMIC emergency care surveillance and registry research. Few articles were from established longitudinal surveillance or registries and primarily composed of short-term data collection. Using these articles, a working group was convened by the US National Institutes of Health Fogarty International Center to discuss challenges and potential solutions for established systems to better understand global emergency care in LMICs. The working group focused on potential uses for emergency care surveillance and registry data to improve the quality of services provided to patients. Challenges included a lack of dedicated resources for such research in LMIC settings as well as over-reliance on facility-based data collection without known correlation to the overall burden of emergency conditions in the broader community. The group outlined potential solutions including incorporating data from sources beyond traditional health records, use of standard clinical forms that embed data needed for research and policy making and structured population-based research to establish clear linkages between what is seen in emergency units and the wider community. The group then identified current gaps in LMIC emergency care surveillance and registry research to form a research agenda for the future.

**Summary box**

- Emergency care surveillance and detailed registries of emergency care are largely absent in most low-income and middle-income countries (LMICs).
- Despite challenges, setting up emergency care surveillance and registry systems can address critical data needs, improve quality of services provided to patients and support public health.
- Potential strategies include incorporating data from sources beyond traditional health records, use of standard clinical forms that embed data needed for research and policy making and structured population-based research.
- Gaps remain in LMIC emergency care surveillance and registry research, and additional work is needed to establish the evidence base and identify effective means of implementation.

## Background

**Figure 1:**
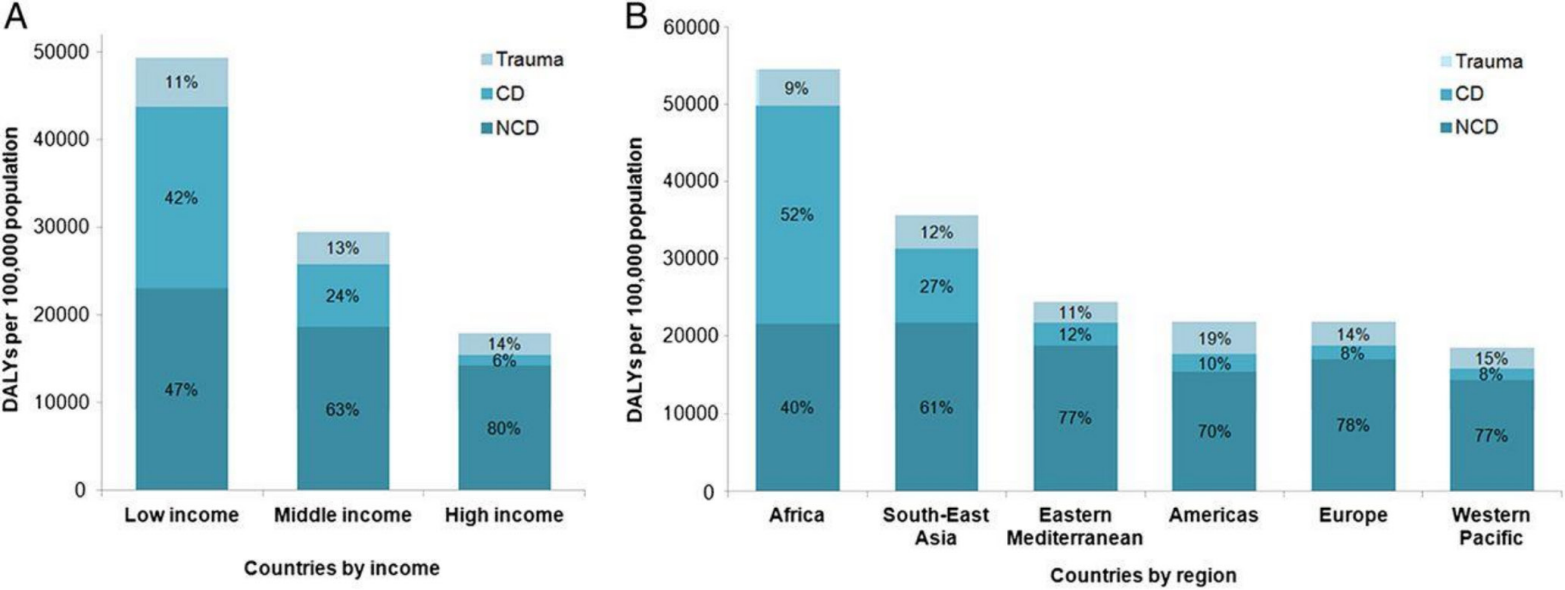
DALYs per 100 000 population attributable to emergency conditions by aetiology: separated by income level (A) and region (B).^2^ CD, communicable disease; DALYs, disability-adjusted life years; NCD, non-communicable disease.

The majority of the world’s population does not have timely access to quality emergency care when emergencies occur.^1 2^ Despite this fact, a major proportion of low-income and low middle-income countries (LMICs) deaths are attributable to conditions that are amenable to emergency care.^2 3^ For the purpose of this paper, emergency medical care constitutes care provided to a patient suffering from acute, potentially life-threatening illness in the first few minutes to hours of care, irrespective of the patients’ location. A recent review demonstrated that ‘all 15 leading causes of death and disability-adjusted life years (DALYs) globally with potential emergent manifestations’ (figure 1) and that the burden of emergency conditions was highest in LMICs.^2^ Many of these patient encounters may never be adequately captured in traditional health information systems.

**Figure 2:**
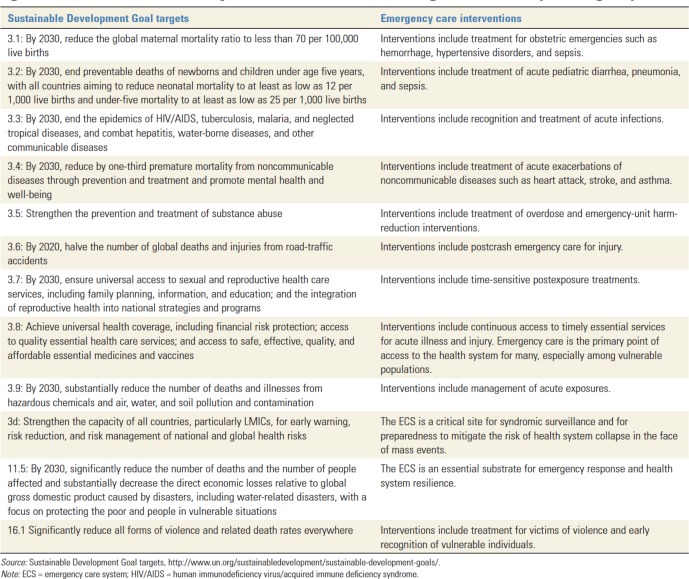
Sustainable Development Goal #3: health, targets affected by emergency care.^5^

As emphasised by Reynolds *et al*, there is a large burden of disease caused by emergency conditions. Furthermore, many important targets of the Sustainable Development Goals (particularly #3 – *Ensure healthy lives and promote well-being at all ages*) are affected by emergency care, including target 3d, which emphasises the critical importance of the emergency care system for syndromic surveillance and preparedness (figure 2).^4–6^ Thus, it is imperative that accurate, reliable and timely information regarding emergency conditions is available for policy makers, especially in LMICs where the burden of emergency conditions is most acute. However, LMIC policy makers face frustration when even basic data on emergency care is limited.^4 7 8^ Current data gaps are both qualitative and quantitative. Many facilities are unable to accurately identify the proportion of acute visits that represent the most severely ill and injured in order to plan their patient care capacity, the number of staff and their levels of training and availability of material resources.^4 7 9^ In addition, little data are available on patients’ motivations for pursuing emergency care in LMICs or their perception of the quality and acceptability of the care they receive. Furthermore, in some LMICs, patients that die in the first 24–48 hours are classified as ‘Brought in Dead’.^10^ Such an approach negates the very *raison d’etre* of emergency medicine as it is *these very patients*—those that survived to presentation—that represent potentially avertable deaths most in need of emergency care.

The objective of this paper is to describe the role of surveillance and registry in strengthening emergency care and public health, especially in LMICs and discuss challenges and potential solutions for establishing such systems. This paper is the result of work conducted by a multidisciplinary working group, which was established under the auspices of the Fogarty International Center at the National Institutes of Health (NIH) as part of the broader Collaborative for Enhancing Emergency Care in LMICs (CLEER). The working group was composed of 10 experts representing 8 countries (5 LMICs), who applied and were selected to participate in the CLEER project based on their expertise and experience with surveillance and registries in LMICs and emergency medicine. The group was split evenly between researchers and practitioners from LMICs and those from high-income countries (HICs) with extensive experience in LMIC emergency care research. The group met physically at NIH for 2 days in July 2017 and then continued to teleconference several times over the next year.

**Figure 3:**
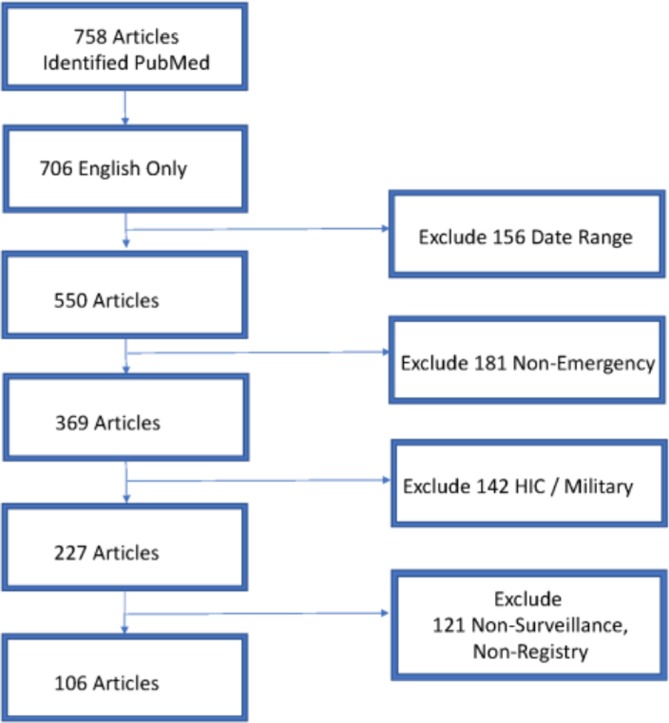
PRISMA diagram of literature search LMIC emergency care registries and surveillance. HIC, high-income countries; LMICs, low-income and low middle-income countries; PRISMA, Preferred Reporting Items for Systematic Reviews and Meta-Analyses.

The working group convened at a workshop and then undertook a literature review regarding the use of surveillance and registries for emergency care in LMICs (figure 3). A search of the PubMed/MEDLINE database keywords related to emergency care system registry, and surveillance research was initially completed by a biomedical librarian at the NIH in February of 2017 in preparation for the July meeting as background material. The search specifically targeted the use of various types of data collection, surveillance systems and registries to collect data on acute care/emergency care in LMICs. LMIC inclusion in the search was based on existing World Bank classifications.^11^ Search results were limited to those published in English over 10 years (from January 2007 to December 2016) based on the recommendation of the NIH librarian to generate a manageable yet comprehensive list of background material that would be accessible to all participants in a common language, which yielded 550 individual results (online supplementary file 1).

10.1136/bmjgh-2019-001442.supp1 Supplementary data

**Figure 4:**
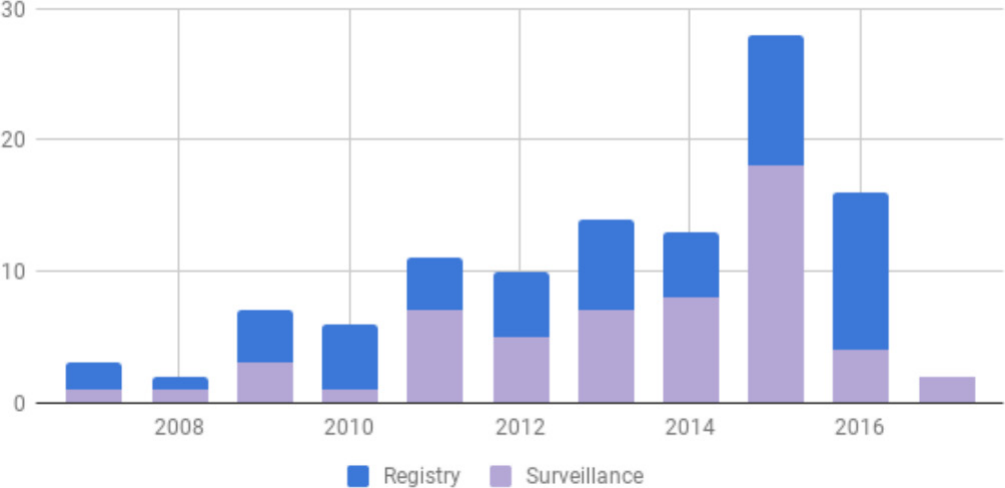
Emergency care surveillance and registry articles in LMICs 2007–2016. LMICs, low-income and low middle-income countries.

After the NIH meeting, the working group decided to refine the literature for inclusion as a component of this manuscript and screened the articles to identify only those articles that: (A) explicitly focused on emergency care (including acute medical, surgical and trauma presentations for all ages); (B) came from LMIC settings (excluding those from military medical units located in LMICs); and (C) resulted from either surveillance or registries (figure 3). The result of the search was 106 articles in total from LMICs that analysed emergency care surveillance or registry data over the 10-year period (figure 4). This includes a large number published in 2015 from the Pakistan National Emergency Departments Surveillance Project (PAK-NEDS) Emergency Care Surveillance Project in Pakistan, which yielded a spike in articles for that year. Articles were reviewed for methodological keys to successful implementation and for barriers to sustainability.

For the purpose of this paper, we define surveillance as an ongoing, systematic collection, analysis, interpretation and dissemination of health information.^12^ Emergency surveillance can provide important information about the epidemiology of emergency conditions, the size and scope of emergency health problems, identification of populations at risk, identification of risk factors, recognition of unusual syndromes (such as new infectious outbreak) as well as tracking the effects of public health interventions. We treat emergency care surveillance data collection as being on a spectrum from *basic* (core data set); *expanded* to highlight those most critically ill and injured for whom a time sensitive intervention by trained emergency care provider may be most needed; and *longitudinal* (registry data that include risk factors, causes of presentation, severity of illness, treatments received and outcomes).

Registry, however, is defined as the process of data collection that incorporates longitudinal data on patient care processes, presentation severity and outcomes allowing for assessment of quality of care and performance of the emergency health system for given conditions. Registries are vital tools for understanding the impact of emergency conditions as well as the ability of the emergency care system to treat them effectively.

In addition, we limit discussion here to emergency surveillance primarily within the health sector while making suggestions for how data from other sectors can inform emergency care planning. Furthermore, we focus on core data collection with reference to prior guidelines such as the WHO Injury Surveillance Guidelines^13^ and the newly updated minimum datasets for emergency care (trauma and non-trauma, 2017).^14–16^ Examples of recent emergency care surveillance and registries were selected for illustration of both best practices as well as challenges faced in implementation of such programme (online supplementary appendix 1 and 2).

## Emergency care system based surveillance and registry

Data regarding the causes, risk factors, clinical care, clinical outcomes and costs of emergencies in LMICs is limited, particularly among those with limited financial or geographical access to emergency care.^4 6–8^ Setting up emergency care surveillance and registry systems can address these critical data needs. Emergency care surveillance can support public health through:

1. Defining the burden of emergency conditions and variations in that burden within countries and between the LMICs.
2. Describing the prevalence of modifiable risk factors.
3. Outlining the processes of care and its impact on access and outcomes.
4. Delineating the severity of clinical presentations and diseases.
5. Evaluating the effectiveness and appropriateness of emergency care treatment protocols in each setting.
6. Evaluating cost-effectiveness of the emergency care interventions.
7. Assessing outcomes of emergency care for both admitted patients and those discharged to the community.

Furthermore, information on the socioeconomic spatial determinants of disease can be obtained by:

1. Linking emergency care surveillance data with other longitudinal and horizontal health data collection systems.
2. Linking emergency care surveillance with non-clinical data systems (eg, police statistics and mortuary records).

Addressing these key areas poses methodological challenges. While randomised trials allow for analysis of specific interventions and exposures, the availability of emergency care *itself* is neither uniformly distributed nor universally accessible in LMICs. This key factor among many others cannot be randomised to assess efficacy of care. Improving emergency care in LMICs will initially rely heavily on observational studies.

## Framework for emergency care surveillance

**Figure 5:**
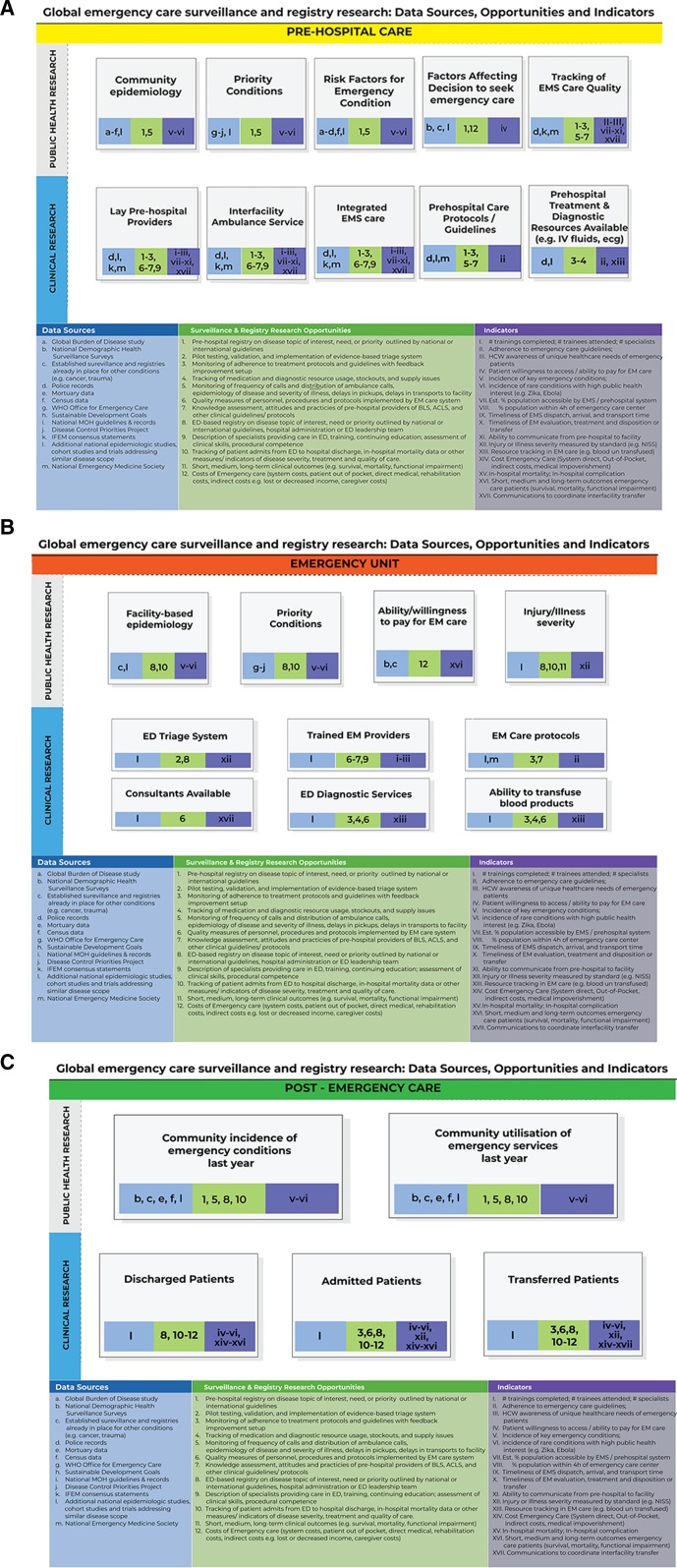
Model for emergency care surveillance and research opportunities: (A) prehospital; (B) emergency unit; (C) postemergency. EMS, Emergency Medical Services; WHO, World Health Organization; MoH, Ministry of Health; IFEM, International Federation for Emergency Medicine; ED, Emergency Department; EM, Emergency Medicine.

We introduce a framework for conceptualising opportunities for surveillance and registry research in low-resource emergency care settings. Dividing emergency care into three phases—prehospital, facility-based emergency care and postemergency care—there are opportunities for both clinical and public health focused research (figure 5). In each of these domains, there are important questions that can inform public health initiatives and improve clinical care, using and expanding on existing data sources. Within these domains, there are further unmet needs for establishing dedicated data collection efforts to fill the gaps about the care and conditions of these patients.

## Emergency care system surveillance: potential sources of data

Emergency care surveillance can take place in many settings based on the location of a patient with an emergency condition and can make use of many sources of health. In addition to facility records, emergency care surveillance can use alternate sources of data such as ambulance records (eg, road traffic injuries),^17^ community surveillance (eg, drowning registry),^18^ as well as data from non-medical sources of data (eg, police records with notations of injuries/fatalities from interpersonal violence),^19 20^ insurance and labour information on workplace injuries^21^ and mortuary records^22^ that provide a cause (even if not precise) of deaths to contribute to understanding emergency care in an LMIC community. Ideal surveillance systems would integrate and harmonise multiple sources of data and allow for analysis of non-health databases to generate information about illnesses and injuries of local interest.

In addition, there is a variety of data collection efforts taking place in dedicated emergency care settings, each with a distinct and important function. Literature primarily from HICs has shown that screening for non-emergency public health conditions such as those with HIV, hepatitis C, hypertension and pregnant helps connect these high-risk patients to appropriate healthcare resource with a potential to improve or mitigate their health condition and prevent future complications. Such efforts frequently are part of vertical programme with dedicated funding (eg, maternal and child health).

In seeking to identify future directions of work in this area, it is important to state what an ideal emergency care surveillance system might look like in terms of its functions and to identify gaps and barriers to implementation for future investigation (figure 5).

### Facility-based clinical documentation

Most LMIC facilities record limited or no data on emergency patient encounters. When recorded, such data are often in paper logs without use of standard nomenclature. This makes characterising the types of emergency care provided difficult to ascertain through simple queries. There remains a critical need for routine emergency care surveillance to better characterise LMIC emergency care and to identify critical unmet needs.

Facility-based data are particularly appropriate for assessing risk factors for emergency conditions, severity of presentations, care processes and outcomes for patients who receive formal emergency care. Analysing both short-term outcomes (eg, facility-based mortality) and long-term outcomes (eg, using follow-up data) can identify patients at high-risk of clinical decline and assess the effectiveness of emergency care strategies. Currently, many emergency care treatment protocols in LMICs implement care strategies developed and validated in HICs. Using registries of emergency care encounters, investigators may assess the appropriateness of translating such protocols to LMIC settings and, when indicated, call into question strategies accepted as dogma in HICs when they prove detrimental, as in the case of the Fluid Expansion as Supportive Therapy (FEAST) trial, which demonstrated greater mortality in low-income countries for children with sepsis who were aggressively resuscitated with intravenous fluids.^23^

Facility-based studies are frequently limited to analysing a single emergency care diagnosis (eg, stroke or acute coronary syndrome). Increasingly, emergency care surveillance in LMICs has transitioned to analysing categories of conditions (eg, non-communicable diseases (NCDs) and injuries). The WHO Stepwise Approach to Surveillance is one such promising approach where a standardised set of questions and protocols is used to allow for monitoring within-country trends as well as to compare surveillance data between countries.^24^ While not exclusively conducted in emergency care units, this approach has been used successfully for surveillance of conditions relevant to emergency care including diabetes, hypertension and stroke as well as to monitor trends in important risk factors such as smoking and obesity in emergency patients.^25 26^

### Population-based health studies

**Figure 6:**
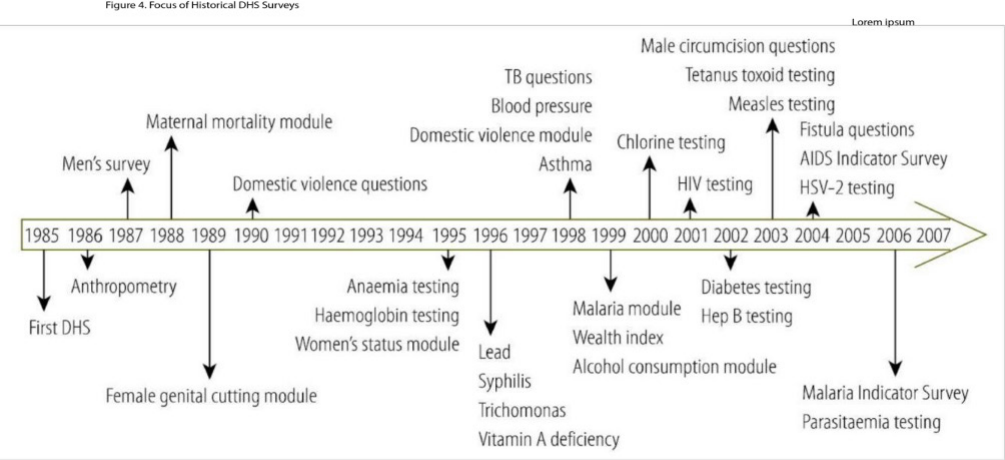
Demographic and Health Survey (DHS) timeline: key survey question, module and biomarker milestones, 1985–2006.^26^

While not considered as traditional surveillance, cross-sectional population-based studies repeated over time are an important source of health data in LMICs, perhaps the best example of which are the Demographic and Health Surveys (DHS) (figure 6). While over 200 DHS studies have been conducted in over 80 countries since 1984,^27^ these studies have focused primarily on infectious diseases, malnutrition and maternal and child health.^28^ Notably, survey indicators often do not contain data on critical emergency presentations such as complications of NCDs and injuries (figure 6).

## Challenges and opportunities for LMIC emergency care surveillance

### Population representativeness of facility-based emergency surveillance

We identified no studies that compare the incidence of emergency conditions among patients who seek emergency care compared with the total population. As such, the limited available data may underestimate or overestimate the prevalence of emergency conditions in LMICs. Additional work may be done to ascertain any relationship between these two groups (emergency patients vs general population) for conditions of significant public health interest in LMICs (eg, trauma in young people (online supplementary appendix 2), acute coronary syndromes (online supplementary appendix 1) and obstetric complications). Defining the level of undercounting (or over counting) using robust epidemiological methods can help correct for the level of the systematic error from emergency surveillance compared with the population-based rates.

### Limited emergency care coverage and its impact on estimating population level rates

Furthermore, while geographic distance may be one obvious barrier to seeking emergency care, few published studies examine other factors that influence patient care-seeking behaviour. A recent article by Ouma *et al* 1 revealed that 29% of people in sub-Saharan Africa are located more than a 2-hour travel from the nearest hospital with marked differences within and between countries (range 25%–90% within 2 hours). Such barriers to accessing emergency care can result in some LMIC patients presenting ‘sicker and later’ than patients presenting with similar conditions in HICs.

### Loss to follow-up and incomplete outcome ascertainment

Many patients in LMICs receive only episodic care. Emergency care surveillance may represent the only opportunity to capture data on these patients in national health statistics. Despite the prime role of emergency care in provision of healthcare to large percentages of patients, in our experience few emergency units have systems in place to track outcomes of patients longitudinally. No data exist to quantify the availability of systems for tracking patient outcomes. High rates of loss to follow-up and incomplete outcome ascertainment make the evaluation of treatment protocols and assessment of the real morbidity and mortality of these patients difficult.

Furthermore, given the poor coverage of emergency care in many LMICs referenced above, it is reasonable to assume that patients for whom follow-up is achieved are not representative of emergency patients on the whole. Attempting to account for this bias with modelling is limited when mortality is a significant cause of loss to follow-up.^29^ The best models for adjusting for this potential large source of bias are from the HIV literature where clinical data have been linked to vital registries to identify loss to follow-up due to mortality in sub-Saharan Africa. In these studies, death accounts for 12%–87% of patients lost to follow-up.^30^

### Role of registries to ascertain meaningful outcomes

Emergency care surveillance is needed to identify causes of presentations and sentinel surveillance for conditions of public health importance in LMICs. Additional work is needed to implement registries in LMIC emergency units to capture critical outcome information for continuous quality improvement and to enable trials of interventions in these populations. As the results of such surveillance and registry data are incorporated into decision making, they can be used by decision makers to design and measure the effectiveness of public health interventions and assess the quality of emergency care.

### Ascertainment of risk factors and misattribution

As many patients seek emergency care for decompensation of chronic conditions, ascertainment of risk factors and complete medical histories are necessary to properly attribute causes of morbidity and mortality. Even with reasonably complete mortality (through linking to vital registries, for example) without adequate emergency care surveillance and registries that account for these risk factors, there is a significant risk of misclassification of the causes that led to patients seeking emergency care. Currently used clinical records do not capture complete data on risk factors and, when captured, do not render them in an analysable form. Furthermore, many risk factors rely on diagnostic testing that is frequently limited in LMIC emergency care units. Self-reported risk factors, especially for stigmatised behaviours, may be severely under-reported (eg, alcohol and substance use) requiring independent testing for accurate assessment.

### Limited capacity for data management

While advances in information technology allow for increasing amounts of data capture, it is understood that most LMIC emergency care centres still operate with paper records and with limited resources for data management. As such, it may be costly and impractical to record data on all emergency presentations in LMICs. In addition, ‘too much data’ creates a ‘signal to noise’ problem, making timely analysis of surveillance difficult and limiting the ability to make timely use of the information obtained from emergency surveillance. Furthermore, implementing data collection as an added unfunded mandate to front-line medical workers quickly result in frustration of clinical staff as well as incomplete data due to lack of timely completion of data collection instruments. This is compounded when the impact of the data collection is not felt by the clinical providers. Timely analysis and feedback of results to clinical staff can reduce these barriers as staff begin to see the impact of data collection and surveillance on their daily work. Examples could include monitoring, coordination and reporting of intensive care unit bed availability or medication stockouts in a region to help plan emergency care.^31^ It is important to consider what conditions are of critical importance to the emergency system in order to prioritise data collection for those cases.

### Emergency care system based surveillance and issues of definitions

What defines a critical condition may vary from one system to another. Decision makers may choose to highlight cases that disproportionately result in mortality, or those that affect certain populations (eg, children), or those that consume a disproportionately high level of resources, especially in resource-limited settings where fixed resources are dedicated to the provision of emergency care. Furthermore, such care is provided whether there are discrete emergency care units or dedicated emergency care practitioners. This makes routine collection of emergency care data more challenging when it is not part of a discrete emergency care unit.

## Proposed solution to emergency care system surveillance and registries

### What should LMIC emergency care surveillance target?

Currently, scholarship is sparse on what cases are most important to include in emergency care registries, and this is an important area for further research. In the absence of locally delineated priorities, it is proposed that initial emergency care surveillance focus on conditions contributing to the largest burden of disease as outlined in national data. These cases can be complemented by cases of public health concern (eg, haemorrhagic fever) where and when they are prevalent.

### Variables to be included in a surveillance system

While there is not an established evidence base for which variables are most important for emergency care surveillance, models do exist (eg, for trauma care) to delineate different levels of detail for data collection.^13^ Similar work needs to be done to establish a core set of variables for emergency care surveillance. Evaluation criteria for developing this set may consider populations of interest, cost to the system, local burden of disease, measures of severity and feasibility of data collection. A suggested initial core set could include:

- Facility descriptors.
- Demographics.
- Chief complaint or reason for visit.
- Measure of severity (eg, vital signs or shock index).
- Provisional emergency diagnosis.
- Disposition.

An expanded set may include (where available):

- Circumstances of presentation.
- Mode of arrival.
- Time to presentation.
- Time from arrival to provider evaluation.
- Time from arrival to emergency care interventions (eg, emergency operating theatre).
- Length of stay to disposition.
- Number of intermediate stages (eg, previous hospital evaluation/transfer).

In some LMIC settings, there are data available on emergency care related events that take place outside of the health facility, collected by other agencies. Proposed emergency care surveillance may include a limited number of these variables such as:

- Number of motor vehicle collisions and deaths at accident scene (police data).
- Causes of death in community (mortuary records or burial licences).

### Prioritising key variables for data collection

An important first step to initiating emergency care surveillance is to ascertain what related data are currently being collected by health facilities, in what format, what the current restrictions are on their use and what resources are currently available for surveillance more generally. In order to develop a parsimonious list of variables collected, a matrix should be made of all variables to be collected that link each variable to the proposed analysis to which they will contribute, the stakeholder to which such an analysis would be directed, the proposed method of collection and the individual responsible for collection. Only variables that meet all criteria should be included and then ranked by key stakeholders and policy makers for priority. Answers to these questions can determine whether there are opportunities to make modular enhancements to existing systems rather than create new standalone systems. This can enhance stakeholder support as well as preserve effort and resources in resource constrained environments.

### Measuring economic cost of emergencies in LMICs

It is proposed that some percentage of analyses be dedicated to economic impacts of emergency care. Most countries have fixed health budgets. Econometric analyses that specifically tie the impact of certain emergency conditions in financial terms are vital to both making the case for resource allocation to policy makers and non-health stakeholders (eg, ministry of finance) as well as to demonstrate the impact interventions in the health system in the saving of resources as well as lives. These calculations can help justify the upfront and running costs of maintaining surveillance and registry systems and are needed to ensure sustainability of these efforts.^5^

### Method for collecting emergency care surveillance and registry data

When possible, utilisation of electronic health records can facilitate rapid extraction, analysis and dissemination of emergency health data. Recognising that not all facilities that provide emergency care will routinely have access to electronic health records, periodic sampling of emergency care data using electronic data collection instruments (such as those built on open platforms like KOBO Toolbox and others) can provide a periodic look at emergency care and allow for near real-time validation of data collected.

Since for the foreseeable future many settings will continue to use paper records for emergency care documentation, the use of standardised clinical documentation instruments with embedded care variables will be key to routine emergency care surveillance. Additional work needs to be done to define core sets of certain variables (eg, chief complaint) that can define a large percentage of presentations and can reduce the use of free-text data entry.^9^

### Operationalising surveillance and registries in low-resource settings

The question of whose responsibility it is to collect emergency care data is not a simple one to answer. While ministries of health may indeed recognise the importance of such surveillance, emergency care cadres are already overtaxed and frequently are forced to provide emergency care with inadequate numbers of personnel (online supplementary appendix 1). Adding additional responsibilities to overworked public sector health personnel as an unfunded mandate presents a critical point of failure in the system (online supplementary appendix 1 and 2). Dedicated personnel for collection and preparation of surveillance data are ideal, but health budgets in many low-income countries preclude adding workers who are not providing health services themselves. While savings may be realised from more effective or efficient emergency care, without evidence of such savings upfront, this ideal will not likely be implemented.

A compromise solution between these imperatives is to use standard clinical documentation tools with key variables important for emergency care surveillance embedded within them. The WHO Emergency Care Office has developed instruments for documentation of trauma cases as well as routine medical cases (online supplementary file 1). The forms were designed to guide even non-specialist practitioners through routine emergency care evaluations. The tools are linked to standard analysis tools to allow for rapid analyses and data visualisations. The tools were developed in a consensus process and are currently being piloted in emergency care settings worldwide.

### Stakeholder inclusion

By its nature, emergency care encompasses care provided for conditions traditionally covered by a variety of other specialties. This professional overlap poses particular challenges for successful implementation of emergency care surveillance. Perhaps the most important challenge to overcome for successful emergency care data collection is the inclusion of partners from other specialties (online supplementary appendix 2). These include both clinical and non-clinical stakeholders with an interest in emergency care data including: emergency care units, prehospital systems, other clinical specialties, public health units, health ministries, finance ministries, non-health ministries with relevant data that intersects emergency care data (eg, ministries of civil defence and planning), community members, local industries, non-governmental organisations and academia.

### From cross-sectional observations to surveillance

Many surveillance and registry efforts are initiated as parts of research or programme evaluation programmes (online supplementary file 1). Many of these are tied to vertical health programmes (eg, HIV and maternal health). Such programmes are also important stakeholders as there may be opportunities through partnerships to leverage such data collection efforts to enhance routine data collection of emergency encounters.

A suggested best practice may be that a transition plan to local stakeholders be made at the outset of all such research data collection efforts. Such a plan should include: what subset of the data being collected is appropriate for routine collection, evaluation of how to incorporate core variables into routine clinical documentation, advocacy for core variables to be adopted for routine collection by the Ministry of Health that could provide a mandate for ongoing data collection and involving a broader group of stakeholders in early (research) phase of data collection and dissemination of results to create constituencies for these data.

### Cross-cutting challenges

Cross-cutting challenges to effective implementation emergency care surveillance remain including the need to develop a culture of data use in many settings. Many decision makers do not have a culture of seeking evidence for decisions or are limited in doing so by other imperatives (eg, politics and urgency of decisions). In addition, emergency care remains fragmented and provided in heterogeneous environments. Without dedicated resources for data collection and analysis, these efforts compete with other pressing needs—most specifically provision of health services (Online supplementary file 1).

## Key opportunities for investigation

Analysis and recommendations presented in this paper and elsewhere are primarily based on the expert opinions of an international working group of physicians and public health practitioners. They have not been validated through empirical investigation. Early areas of inquiry should focus on validating proposed methods to assess their ability to characterise the emergency conditions and to impact quality of emergency care.

Furthermore, there is still a need to assess the ability of emergency care surveillance to:

- Approximate the burden of acute illness and injury that exists in the general population (eg, through capture–recapture studies).
- Identify the proportion that results in care seeking behaviour on the part of the community (eg, via comparative facility and population based studies).
- Assess the ability of emergency care surveillance to accurately and reliably identify epidemiological changes in the health of communities (eg, through routine epidemiological surveillance in emergency care centres).
- Identify outbreaks of diseases with public health importance (eg, through syndromic surveillance).
- Better characterise the prevalence of risk factors (such as diabetes and hypertension) in otherwise healthy patients who use emergency centres for episodic care as their only routine healthcare.

Through emergency care surveillance, it may be possible to identify causes of early or prehospital deaths and to identify delays in the chain of survival. Such surveillance can further identify poorly served areas or populations at special risk as well as to benchmark clinical performance for index conditions across facilities, regions or countries (eg, for sepsis, acute coronary syndromes or acute respiratory distress).

There is work to be done to assess the best methods to incorporate data from vertical health programme (eg, for HIV) as well as non-health data (eg, police and mortuary records) into routine emergency care surveillance. Importantly, some conditions may be more representative of the performance of the *emergency care system* itself, like respiratory distress from asthma or major trauma with haemorrhage where time-sensitive interventions characterise effective care.

Implementation science efforts are needed to test the validity, reliability and utility of new surveillance instruments, including piloting of instruments developed by the WHO for emergency care clinical documentation that incorporate variables needed for surveillance. In addition, more work is needed to identify which factors are necessary to successfully generalise models of emergency care surveillance to emergency care settings in different LMICs.

### Limitations

There are several limitations to consider when evaluating the analysis and recommendations presented in this paper. This body of work is largely directed by a panel of individual expert opinions, and the literature was not evaluated as part of a systematic review. The working group met physically once, and then continued to engage remotely, which somewhat limits the free flow of information. The working group itself was initially only 11 people, from 8 different countries. The group whittled down to 10 during the year, somewhat concentrating the bias but fortunately maintaining the LMIC representation and perspectives from Africa, Asia and South America. Additionally, the majority of group members were emergency medicine clinicians, thus the group as whole could have been more representative of other views.

## Conclusion

In the opinion of the working group, emergency care surveillance as well as detailed registries of emergency care are largely absent in most LMICs. The lack of such systems represents an important gap in our understanding about the health of large portions of the population that only receive episodic care, what conditions and populations drive emergency care presentations and costs, as well as the quality of emergency services when they are provided. Additional work is needed to establish the evidence base for different types of emergency care surveillance and to identify effective means of implementation of surveillance and registry systems that are adaptable to a variety of settings including resource-constrained environments.
