# Correction: *Emergency care surveillance and emergency care registries in low-income and middle-income countries: conceptual challenges and future directions for research*

**DOI:** 10.1136/bmjgh-2019-001442corr1

**Published:** 2019-09-19

Mowafi H, Ngaruiya C, O'Reilly G, *et al*. Emergency care surveillance and emergency care registries in low-income and middle-income countries: conceptual challenges and future directions for research. *BMJ Global Health* 2019;4:e001442. doi: 10.1136/bmjgh-2019-001442

The authors want to alert the readers on the incorrect affiliation published for the co-author Andres M Rubiano which is now updated in the published version.

