# Supplementary material for: Emergency care surveillance and emergency care registries in low-income and middle-income countries: conceptual challenges and future directions for research
Source: BMJ Glob Health. 2019 Jul 29;4(Suppl 6):e001442. doi: 10.1136/bmjgh-2019-001442 (PMC6666805; doi:10.1136/bmjgh-2019-001442)
Supplement: Supplementary data [file bmjgh-2019-001442supp001.pdf]

Appendix 1 – Emergency Care Surveillance – Case of the Pan-Asian Resuscitation Outcomes Study (PAROS)

Background

The Pan-Asian Resuscitation Outcomes Study (PAROS) is an out-of-hospital cardiac arrest (OHCA) clinical research network (CRN) comprising Asia-Pacific countries, including Japan, Korea, China, Taiwan, Singapore, Malaysia, Thailand, India, Pakistan, Philippines, UAE-Dubai, and Vietnam. The PAROS network was established in 2010 to understand the burden of OHCA and identify gaps in prehospital and hospital systems in the Asia-Pacific. Key stakeholders from seven countries, A/Prof. Marcus Ong (Singapore), Prof. Sang Do Shin (Korea), Prof. Hideharu Tanaka (Japan), Prof. Matthew Ma (Taiwan), A/Prof. Nik Hisamuddin (Malaysia), Dr. Pairoj Khruekarnchana (Thailand), and Dr. GY Naroo (UAE-Dubai), collaborated to conceive and implement PAROS in their respective country. The PAROS study coordinating centre (SCC) was set up in Singapore, with the support from Mr. Damien Tan and the late Dr. Khoo Teng Chuan from the Singapore Clinical Research Institute (SCRI). Technical advice was provided by the Cardiac Arrest Registry to Enhance Survival (CARES) led by A/Prof. Bryan McNally, in consultation with Prof. John Rush from Duke-National University of Singapore Medical School. The agenda of the PAROS CRN was discussed by all stakeholders involved the following objectives:

1. Describe current pre-hospital systems in the Asia-Pacific;
2. Provide international benchmarking and study of best practices;
3. Impact community awareness and change attitudes towards OHCA;
4. Improve OHCA survival by system/ community level interventions.

Implementation phase

The implementation of PAROS CRN was not without significant challenges, including funding, stakeholders' buy-in, and technical limitations.

Funding

Each participating country/site has to seek their own funding to support research administrative expenses, including manpower for data collection and some sites were unable to secure such funds. The PAROS SCC had tried to assist by securing funds from philanthropic organizations such as Laerdal Foundation and Ramsey Social Justice Foundation to support manpower and travel expenses for low-middle income countries. The SCC had also supported these countries by sponsoring their travel expenses (flight and accommodation) for annual executive committee meetings through a national level grant awarded to the PAROS chair (A/Prof. Marcus Ong).

Buy-in from stakeholders

Countries with existing registries encountered difficulties getting buy-in from e.g. government agencies in submitting data to the registry. There were also challenges establishing collaboration with emergency medical services (EMS) agencies and hospitals, resulting in incomplete representation of OHCA in a city/state/country.

Technical limitations
Technical support and consultation were provided by CARES, Sansio and the SCRI for the electronic data capture (EDC) system. The process of establishing the system was challenging due to time differences, communication channels (in-person communication was not possible), etc.

Operational phase

Data entry is done using the EDC system, an online data registry system that was set up with technical assistance from CARES and Sansio. Emergency medical services data are collected from EMS dispatch and ambulance personnel. Emergency medical services timings are automatically recorded by the respective dispatch systems, with computerized system timings where available. Prospectively collected data are verified by local coordinators before and after entry into the EDC. Each participating site or EMS system has a designated local coordinator, who is responsible for ensuring the accuracy of data entry and verifying records. In addition, each local coordinator must respond to any data queries or verification requests from the SCC within 2 weeks.

The EDC has inherent quality assurance checks and validations. This includes in-built validation rules that cross-check data fields, as well as mandatory fields that must be filled to complete a case. The system will also flag missing fields for the coordinator's verification. In addition, the SCC maintains a stringent data quality review and audit process including verification with local coordinators and source documents. All data entries go through two levels of screening, at the local and SCC levels.

Data contributed by participating countries were analyzed by epidemiologists or biostatisticians from the SCC. Results from the combined data analysis have been presented in regional and international conferences. Each country's participating investigators were given the dataset (full dataset from all countries and country-specific dataset) cleaned by the SCC for reporting to their hospital or to their health ministry. All site principal investigators were also given summary and Utstein reports generated either by the EDC or the SCC on a yearly or ad-hoc basis.

Outcome phase

PAROS registry is currently ongoing and is in the analysis stage for phase 2 of the project. Findings from Phase 1 of the study were published in international peer-reviewed journals such as Resuscitation, Annals of Emergency Medicine, etc. A list of publications can be found at the PAROS CRN's webpage: https://www.scri.edu.sg/crn/pan-asian-resuscitation-outcomes-study-paros-clinical-research-network-crn/paros-publications/

Maintenance of the PAROS CRN
The SCC and the network's investigators face similar challenges as during the implementation phase for their continued participation in the PAROS CRN.

Funding

The SCC faces high manpower and administrative costs as it is responsible for not just data management but the management and disbursement of funds to participating sites as well. The sustainability of the CRN can be threatened in the absence of long-term funding to support the team of research personnel for data collection and management.

Lack of interest from stakeholders

Resistance in collaboration from relevant departments or organisations is still evident in some participating sites, resulting in partial representation of OHCA.

Technical challenges

Manual data entry is still required, and participating sites have faced challenges acquiring manpower to perform this duty. The PAROS EDC is unable to auto-populate data through linkages to the EMS or hospital database.

Appendix 2 – Emergency Care Registry – Case of the Pakistan National Emergency Department Surveillance System (PAK-NEDS)

(Pak-NEDS) was a surveillance system implemented in seven Emergency Departments (EDs) across Pakistan.[1,2] The surveillance system ran for four months from November 2010 to March 2011, and was conducted in five different cities: Karachi, Rawalpindi, Lahore, Islamabad and Peshawar. More than 270,000 unique cases were obtained.[2]

Objective

The primary objective of the surveillance system was to capture all encounters presenting to the EDs during the data collection period in order to describe epidemiology of disease, characteristics of presentations and patient outcomes.[1] The secondary objective was to assess feasibility of implementing the system. Lack of data in the emergency care setting acts as a barrier to provision of quality care, allocation of resources, and policy development.[3,4,5] Without primary data, it is impractical to set indicators or to measure quality.[2,3,6] Furthermore, neglecting those in the acute or critical phases of disease, with the majority of care for these patients taking place in the ED, cripples the success of a health system.[3,4,6] The Pak-NEDS study was an incipient step to addressing this data gap on emergency care in Pakistan, the first of its kind in the country.

Stakeholders

The key stakeholders in developing and implementing this system were the Aga Khan University (AKU), a private tertiary care institution located in Karachi, Pakistani, and Johns Hopkins University. The effort was supported by directors of the respective EDs involved and the Society of Emergency Physicians Pakistan.

Process

Data collectors were positioned at each of the EDs in order to capture the information for entry into the system. They used paper forms to collect the data, which were then sent to a coordinating office. A data supervisor reviewed the forms for any errors and sent these into the data coordinating center at AKU on a weekly basis. The data was then entered into a computer database.

Several efforts were made for quality assurance at different phases of the system. Field visits were made by the primary research team to ensure accuracy of data collection through direct supervision of data collectors. Additionally, the field supervisor made random checks on the data collection forms at the collection sites and coordinating center. Finally, double data entry was used when transferring information from the written forms into the computer database.

Outcomes

Outcomes from the surveillance system were research-oriented in scope. Study findings included: the need for optimization of and greater resource allocation for pre-hospital

services;[1] a significant burden of intentional overdose, constituting around half of all poisonings, stressing importance of mental health care; characterization of traumatic brain injury with a predominance of road traffic accidents as cause, and finally, on pediatric presentations with around 40% of these resulting from injury, which is a novel finding in this vulnerable population. Results were distributed to the hospitals from which the Pak-NEDS data was sourced.

Challenges

Challenges faced in sustaining the surveillance system included the volume of patients, which exceeded capacity of data capture methods used. The paper forms presented a limiting factor as they were difficult to keep track of, and made data entry challenging due to illegible handwriting, misspellings and data entry errors. Lack of funding and stakeholder buy-in presented additional insurmountable barriers, specifically lack of engagement from the hospitals involved and ministry of health. Finally, there was a lack of trained personnel to ensure continuity of the system.

References

1. Puvanachandra P, Razzak JA, Hyder AA. Establishing a national emergency department surveillance: an innovative study from Pakistan. *BMC Emerg Med* 2015;15(Suppl 2):I1.

2. Mir M, Bachani A, Khawaja H, et al. The Pakistan national emergency department surveillance study (Pak-NEDS): introducing a pilot surveillance. *BMC Emerg Med* 2015;15(Suppl 2):S1.

3. Hsia R, Razzak J, Tsai A, et al. Placing emergency care on the global agenda. Ann Emerg Med 2010;56:142-49.

4. Kobusingye O, Hyder A, Bishai D, et al. Emergency medical systems in low- and middle-income countries: recommendations for action. *Bull World Health Organ* 2005;83:626-31.

5. Razzak J, Kellermann A. Emergency medical care in developing countries: is it worthwhile? *Bull World Health Organ* 2002;80:900-05.

6. Laxminarayan R, Mills A, Breman J, et al. Advancement of global health: key messages from the Disease Control Priorities Project. *Lancet* 2006;367:1193-1208
